# Reduced expression of alanyl aminopeptidase is a robust biomarker of non‐familial adenomatous polyposis and non‐hereditary nonpolyposis colorectal cancer syndrome early‐onset colorectal cancer

**DOI:** 10.1002/cam4.5675

**Published:** 2023-02-07

**Authors:** Ye Jin Ha, Yun Jae Shin, Ka Hee Tak, Jong Lyul Park, Jeong Hwan Kim, Jong Lyul Lee, Yong Sik Yoon, Chan Wook Kim, Seon Young Kim, Jin Cheon Kim

**Affiliations:** ^1^ Asan Institute for Life Sciences Asan Medical Center Seoul South Korea; ^2^ Personalized Genomic Medicine Research Center Daejeon South Korea; ^3^ Korea Bioinformation Center Korea Research Institute of Bioscience and Biotechnology (KRIBB) Daejeon South Korea; ^4^ Department of Bioinformatics University of Science and Technology (UST) Daejeon South Korea; ^5^ Department of Surgery, Asan Medical Center University of Ulsan College of Medicine Seoul South Korea

**Keywords:** ANPEP, biomarker, early‐onset colorectal cancer (EOCRC), late‐onset colorectal cancer (LOCRC)

## Abstract

**Background:**

Early‐onset colorectal cancer (EOCRC) has been increasing in incidence worldwide but its genomic pathogenesis is mostly undetermined. This study aimed to identify robust EOCRC‐specific gene expression patterns in non‐familial adenomatous polyposis (FAP) and non‐hereditary nonpolyposis colorectal cancer syndrome (HNPCC) EOCRC.

**Method:**

We first performed gene expression profiling analysis using RNA sequencing of discovery cohort comprised of 49 EOCRC (age <50) and 50 late‐onset colorectal cancer (LOCRC) (age >70) specimens. To obtain robust gene expression data from this analysis, we validated differentially expressed genes (DEGs) through TCGA cohort (EOCRC:59 samples, LOCRC:229 samples) and our validation cohort (EOCRC:72 samples, LOCRC:43 samples) using real‐time RT‐PCR. After the validation of DEGs, we validated the selected gene at protein levels using Western blotting. To identify whether genomic methylation regulates the expression of a particular gene, we selected methylation sites using The Cancer Genome Atlas (TCGA) datasets and validated them by pyrosequencing in our validation cohort.

**Results:**

The EOCRC patients included in this study had significantly more prominent family history of cancer than the LOCRC patients (23 [46.9%] vs. 13 [26%], *p* = 0.050). Alanyl aminopeptidase (ANPEP) was significantly downregulated in the EOCRC tissues (FC = 1.78, *p* = 0.0007) and was also commonly downregulated in the TCGA cohort (FC = −1.08, *p* = 0.0021). Moreover, the ANPEP mRNA and protein expression levels were significantly downregulated in the EOCRC tissues of our validation cohort (*p* = 0.037 and 0.027). In comparisons of the normal and tumor tissues in public datasets, the ANPEP level was significantly lower in the tumor tissue in the TCGA dataset (*p* < 2.2 × 10^−16^) and GSE196006 dataset (*p* = 0.0005). Furthermore, the ANPEP expression level did not show a decreasing tendency at a young age in the normal colon tissue of the GTEx dataset. Lastly, the hypermethylation of cg26222247 in ANPEP was identified to be weakly associated with reduced ANPEP expression in our EOCRC cohort.

**Conclusion:**

The reduced expression of ANPEP was identified as a novel biomarker of non‐FAP and non‐HNPCC EOCRC.

## Graphical abstract

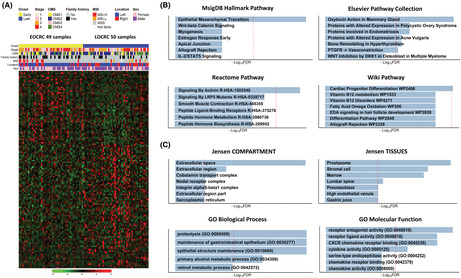

## 1 INTRODUCTION

The global incidence of colorectal cancer (CRC) is not only the third highest among cancers worldwide, but it was also a leading cause of cancer deaths in 2018.^1^ However, although the overall incidence and deaths from CRC have declined since 1998 in the United States,^2^ early‐onset CRC (EOCRC) defined by an age of onset below 50, has been increasing.^3^, ^4^, ^5^ In South Korea, the EOCRC incidence has increased rapidly, whereas the rates of CRC have risen at a similar pace among adults 50 years and older. Neither the etiology of EOCRC nor the reasons for its increasing trend are currently well determined. Although recent studies have been conducted to find genomic variants of EOCRC on a large scale using targeted sequencing data, no genomic tumor differences have been found that can distinguish EOCRC from late‐onset CRC (LOCRC).^6^ Some studies have reported that EOCRCs have different pathological and molecular features from LOCRCs.^7^, ^8^ Of note, in particular, previous research based on RNA sequencing data have reported distinct gene expression patterns in EOCRC.^9^, ^10^, ^11^ In terms of risk factors for EOCRC, westernized diets, processed and red meat, obesity, and high‐fructose corn syrup have been commonly described, and all are known to have negative impacts on inflammation and the microbiome^12^, ^13^, ^14^ that are widely recognized as major drivers of colorectal carcinogenesis.^15^ With these distinct characteristics of EOCRC, it becomes necessary to separately characterize these tumors from LOCRC. Four distinct molecular subtypes of CRC based on gene expression profiling were previously proposed to characterize CRC, known as the consensus molecular subtypes (CMS).^16^ They comprise CMS1 (microsatellite instability and immune), CMS2 (canonical), CMS3 (metabolic), and CMS4 (mesenchymal). CMS1, which is the “immune hot type” is a more prevalent subtype among EOCRCs.^17^, ^18^ However, no clear association of CMS with EOCRCs versus LOCRCs can yet be identified, as CMS classifications have limitations, that is, they are mainly confined to microsatellite instability (MSI), mesenchymal cells, and specific driver gene mutations.^13^

We have here identified the molecular characteristics of EOCRCs and also EOCRC‐specific gene expression, using RNA sequencing analysis. The tumor tissue samples used to derive these data were obtained from 49 EOCRC patients and 50 LOCRC patients. To identify robust EOCRC‐specific gene expression patterns, we validated our sequencing results in the data from The Cancer Genome Atlas (TCGA) colon adenocarcinoma (COAD) and rectum adenocarcinoma (READ) cohorts. We further validated these e‐specific genes in our validation cohort using real‐time RT‐PCR. Our analyses provide new insights into the characteristics of EOCRCs and their specific gene expression profiles.

## 2 MATERIALS AND METHODS

### 2.1 Patient enrolment and sample acquisition

This study was approved by the Institutional Review Board (IRB) of Asan Medical Center (IRB no. 2019–1367). The study subjects had been diagnosed with CRC at Asan Medical Center, Seoul, Korea between 2008 and 2017. Exclusion criteria included a diagnosis of familial adenomatous polyposis (FAP) syndrome or hereditary non‐polyposis colorectal cancer (HNPCC) syndrome meeting the Amsterdam criteria, or the receipt of preoperative chemo/radiotherapy. All samples were stored in liquid nitrogen prior to use. Patients under 50 years (hereafter referred to as EOCRC cases) and over 70 (LOCRC cases) were used in the comparisons. Tumor tissue samples of 49 EOCRC and 50 LOCRC cases were comprised for whole transcriptome sequencing (WTS) analysis. WTS data of tumor tissue samples of 14 EOCRC and 10 LOCRC our previous study (GSE132024) were added to this discovery cohort. In the validation cohort, mRNA expression of tumor tissue samples from 72 EOCRC and 43 LOCRC cases was analyzed by real‐time RT‐PCR analysis.

### 2.2 Bulk RNA sequencing and data analysis

RNA was purified from the tumor tissue samples using the AllPrep DNA/RNA Mini Kit (Qiagen). The concentration and purity of the extracted RNA were measured with NanoDrop and Bioanalyzer (Agilent Technologies). We constructed mRNA sequencing libraries of our dataset using a TruSeq Stranded mRNA LT Sample Prep Kit and the sequencing was then conducted using the Illumina platform. The sequencing reads had a length of 100 bp and were paired‐end. These reads were aligned to the hg38 human reference genome^19^ using HISAT2 aligner version 2.1.0.^20^ The aligned reads were counted using featureCounts in the Subread 2.0.3 package.^21^ Read counts for every gene were normalized with the trimmed mean of M‐values (TMM) method using the edgeR package in R.^22^ We then compared gene expression between the early and late groups to identify differentially expressed genes (DEGs) using the quasi‐likelihood generalized linear model (GLM) of the edgeR package.^22^ DEGs were selected in accordance with the following criteria: *p* < 0.01, |log2FC| (logarithmic fold change of the gene expression) >1, logCPM (logarithmic counts per million reads) > 1. For visualization of the DEGs, we sorted these candidates using the fold change values and plotted a heatmap using complexheatmap package in R.^23^ Overlapped DEGs were shown in a volcano plot, generated using EnhancedVolcano package in R.^24^

### 2.3 Pathway and gene ontology analysis

EnrichR analysis^25^, ^26^, ^27^ of the identified DEGs was used to identify significantly enriched pathways and ontologies in our dataset. These pathways and ontologies were selected under a 0.05 false discovery rate (FDR).

### 2.4 Public data acquisition and processing

TCGA‐COAD and TCGA‐READ datasets, obtained from the GDC portal web page (https://portal.gdc.cancer.gov/), were utilized to validate selected DEGs.^28^ The samples of 59 EOCRC and 229 LOCRC in TCGA‐COAD and TCGA‐READ were used to validate the DEGs. To identify DEGs between the early and late onset CRCs in the TCGA datasets, gene read counts files were obtained for the analysis and were normalized with TMM method of edgeR and then were used as input of analysis. We also obtained gene read counts files from 44 normal tissue samples from the TCGA datasets and normalized with TMM method of edgeR for comparisons between tumor and normal tissues. To validate the gene expression levels of the normal tissue in the TCGA datasets, we used GTEx (Genotype‐Tissue Expression, https://gtexportal.org) v8 RNA sequencing data.^29^ Among the 49 normal tissue types, colon tissue samples (*n* = 555) were obtained with gene read counts. The gene expression levels were calculated with the TMM normalization of edgeR and used for the kendall's rank correlation analysis. For further validation in external dataset, we obtained transcriptome profiling data from GEO's GSE196006 comprised of 21 EOCRC and its adjacent normal tissue. The raw read counts files in GSE196006 were processed with the TMM normalization of edgeR and then used for validation by paired Wilcoxon test. Additionally, the TCGA dataset from the GDC portal were utilized to select candidate methylation site for validation in our cohort. We obtained the methylation beta value data of methylation array of Illumina human methylation 450 K in the TCGA datasets. A total of 355 tumor tissue samples and 43 normal tissue samples in the TCGA datasets were used to identify the methylation status of the selected genes.

### 2.5 CMS subtyping

We used the CMS classifier package^16^ to demarcate the CMS subtypes based on the RNA sequencing results from our discovery cohort. We used the random forest algorithm with the log2 scaled RNA sequencing dataset to identify CMS subtypes. To maximize the number of classified samples, we used the nearest CMS values for our dataset.

### 2.6 Real‐time RT‐PCR

Total RNA was extracted from all of the study patient samples, and from the included cell lines, using the RNeasy Mini Kit (Qiagen). For real‐time RT‐PCR, cDNA was synthesized from these total RNA preparations using random primers and SuperScript II RT (Thermo Fisher Scientific). The amplifications were then conducted on a Roche LightCycler 96 (Roche) with SYBR Green I Master Mix. The primers used to amplify target genes are listed in Table 1. The PCR amplification conditions were as follows: a preincubation at 95°C for 10 min followed by 45 cycles at 95°C for 10 s, 60°C for 10 s, and 72°C for 10 s; melting at 95°C, 65°C, and 97°C for 10 s each; and cooling at 37°C for 30 s. The gene encoding glyceraldehyde 3‐phosphate dehydrogenase (GAPDH) was used as an internal control.

**TABLE 1:** Real‐time RT‐PCR primers for the 10 common DEGs identified in EOCRC.

Genes	Sequence	RefSeq
ANPEP	Forward: GCTGTTTGACGCCATCTCCTAC	NM_001150
	Reverse: GTTCTGGTAGGCAAAGGTGTGG	
CCL19	Forward: CGTGAGGAACTTCCACTACCTTC	NM_006274
	Reverse: GTCTCTGGATGATGCGTTCTACC	
CHGB	Forward: ACCAGACAGTCCTGACAGAGGA	NM_001819
	Reverse: TAACAGTGCCCACCGCTCCAAT	
CPS1	Forward: CTAGCCTGGATTACATGGTCACC	NM_001875
	Reverse: CCTCAAAGGTACGACCAATAGCC	
DKK4	Forward: CGTTCTGTGCTACATGTCGTGG	NM_014420
	Reverse: GTGTGCCATCTTGCTCATCAAGC	
GLDC	Forward: GCTTGGTGAGAATGATGCCTGG	NM_000170
	Reverse: CAGATGTTGCTGGTAGCCTTGTC	
MAP9	Forward: AGAGCATCCAGTGCATCTGCCA	NM_001039580
	Reverse: CTGTGAAGGTTTTTGGTCCAAGAC	
NTS	Forward: CAGCAGGGCTTTTCAACACTGG	NM_006183
	Reverse: CTCATACAGCTGCCGTTTCAGAA	
PRSS33	Forward: ATTGTGCTGCCTGGGAGTCTGT	NM_152891
	Reverse: CAGGACCCAGCTCCCAGACTG	
WASF3	Forward: ACCGATGGCTCCAGCAGACTAC	NM_006646
	Reverse: GCTGACGAAGGCAGTTTGTGCT	

### 2.7 Western blotting

Protein concentration was quantified using Bradford solution (Bio‐Rad). Proteins were resolved by SDS‐PAGE, and then transferred to polyvinylidene difluoride membrane (Millipore). The membranes were incubated consecutively with primary and secondary antibodies. Specific complexes were detected using the SuperSignal West Pico kit (Thermo Fisher Scientific). The following antibodies were used: anti‐alanyl aminopeptidase (ANPEP; Santa Cruz) and anti‐β‐actin, anti‐mouse IgG, and anti‐rabbit IgG from Bethyl Laboratories.

### 2.8 Survival analysis

Survival data were extracted from the Human Protein Atlas website: https://www.proteinatlas.org/ENSG00000166825‐ANPEP/pathology/colorectal+cancer/COAD (COAD, low expression [*n* = 171] and high expression [*n* = 267]) and https://www.proteinatlas.org/ENSG00000166825‐ANPEP/pathology/colorectal+cancer/READ (READ, low expression [*n* = 46] and high expression [*n* = 113]).^30^ The survival status was analyzed in each group using the Kaplan–Meier method and compared by the log‐rank test.

### 2.9 Gene network analysis

To investigate upstream regulators of a candidate gene, QIAGEN Ingenuity Pathway Analysis (QIAGEN IPA Inc., https://digitalinsights.qiagen.com/IPA)^31^ was used to search for gene networks associated with the DEGs identified from our RNA sequencing data. Fold‐changes, *p*‐values, CPM, and FDR were included in this analysis.

### 2.10 Pyrosequencing analysis for methylation status

The DNA methylation status of the four CpG sites was determined by pyrosequencing using genomic DNA from the tissue samples, purified with an AllPrep DNA/RNA Mini Kit (Qiagen). Briefly, genomic DNA (1 ug each) from each of the tissue samples was treated with sodium bisulfite using the EZ DNA methylation kit (Zymo Research). This converted DNA was then used as a PCR template. Primers were designed using Pyrosequencing Assay Design Software (Biotage) for the target CpG sites. All of the primers and PCR conditions for these analyses are listed in Table 2. Pyrosequencing was performed with PSQ HS 96 Gold single‐nucleotide polymorphism reagents on a PSQ HS 96 pyrosequencing machine (Biotage), which quantitatively measures the methylation status of CpG sites. DNA methylation values calculated with this system ranged from 0 (completely unmethylated cytosines) to 100 (completely methylated cytosines).

**TABLE 2:** Primers used for pyrosequencing and amplification conditions used for the ANPEP gene.

CpG site	Primer sequence 5′–3′	*T*_m_, °C	Size, bp
cg19405555	Forward: AAGGAAAAAGAAAAAAATGAGAAG	58	324
	Reverse: biotin‐TAAAATACACCAAAACTCCTACTA		
	Sequencing: GTATTAAGAAAGTTGAATTG		
cg22710306	Forward: GGAGGAGTTTTGGGGGGTTT	58	171
	Reverse: biotin‐TCCCTACCCCTCCAACACTAAACT		
	Sequencing: GGTTATTTTTTTTTAAAAAG		
cg06963233	Forward: GGGAAAGGAGGAGTTTAGTGTTG	58	266
	Reverse: biotin‐TCCCTAACCCTCAATTTACCC		
	Sequencing: GGTTTAGGTAAGTGG		
cg26222247	Forward: TGTGGGGTTTTTGGTTAATATTG	58	120
	Reverse: biotin‐TAAACACCTAAAATTCCCCTTCCT		
	Sequencing: TTTTTTTTAATTTTAGATTT		

### 2.11 Statistical analysis

Statistical comparisons were performed using GraphPad Prism7.0 (GraphPad Software) and R software (version 4.2.1). Data were expressed as mean ± SD. *p*‐Values <0.05 were considered to indicate statistical significance.

## 3 RESULTS

### 3.1 Clinical landscape of early and late‐onset CRC

To identify DEGs between EOCRCs and LOCRCs in our discovery cohort, we classified a CRC patient aged under 50 years at diagnosis as EOCRC (*n* = 49) and over 70 years as LOCRC (*n* = 50). To assess the clinical features associated with the EOCRC group, we applied the chi‐square test for each clinical feature in relation to disease onset (Table 3). Only a family history was found to be significantly related to the onset of CRC in this evaluation. EOCRC patients had a more prominent family history of CRC than LOCRC patients. In addition to comparing the clinical information between the two groups, we classified CRC patients in accordance with their CMS using RNA sequencing data.^16^ CMS1 and CMS3 were more frequent in EOCRC patients than LOCRC patients, while CMS2 and CMS4 were more frequent in LOCRC patients than EOCRC patients. However, while the CMS classification was slightly different in both groups, the proportions of these different subtypes were not statistically significant in terms of distinguishing the onset of CRC.

**TABLE 3:** Clinicopathological characteristics of the 99 enrolled patients with colorectal cancer (CRC).

	Early‐onset CRC	Late‐onset CRC	*p‐*value
Onset age	<50	>70	
Number	49	50	
Sex			0.368
Male	25 (51%)	31 (62%)	
Female	24 (49%)	19 (38%)	
Survival status			0.088
Alive	39 (20%)	30 (60%)	
Dead	10 (80%)	20 (40%)	
Stage			0.594
I	1 (2%)	3 (6%)	
II	15 (30.6%)	19 (38%)	
III	16 (32.7%)	13 (26%)	
IV	17 (34.7%)	15 (30%)	
Tumor location			0.158
Left	36 (73.5%)	29 (58%)	
Right	13 (26.5%)	21 (42%)	
Cancer family history (in fourth degree)			0.050
Yes	23 (46.9%)	13 (26%)	
No	26 (53.1%)	37 (74%)	
MSI status			0.716
MSI	11 (22%)	8 (16%)	
MSS	37 (76%)	41 (82%)	
Not done	1 (2%)	1 (2%)	
CMS subtype			0.568
CMS1	10 (20.4%)	6 (12%)	
CMS2	16 (32.7%)	21 (42%)	
CMS3	11 (22.4%)	9 (18%)	
CMS4	12 (24.5%)	14 (28%)	

### 3.2 Pathway and gene ontology analysis of DEGs between early and late‐onset CRC

**FIGURE 1:**
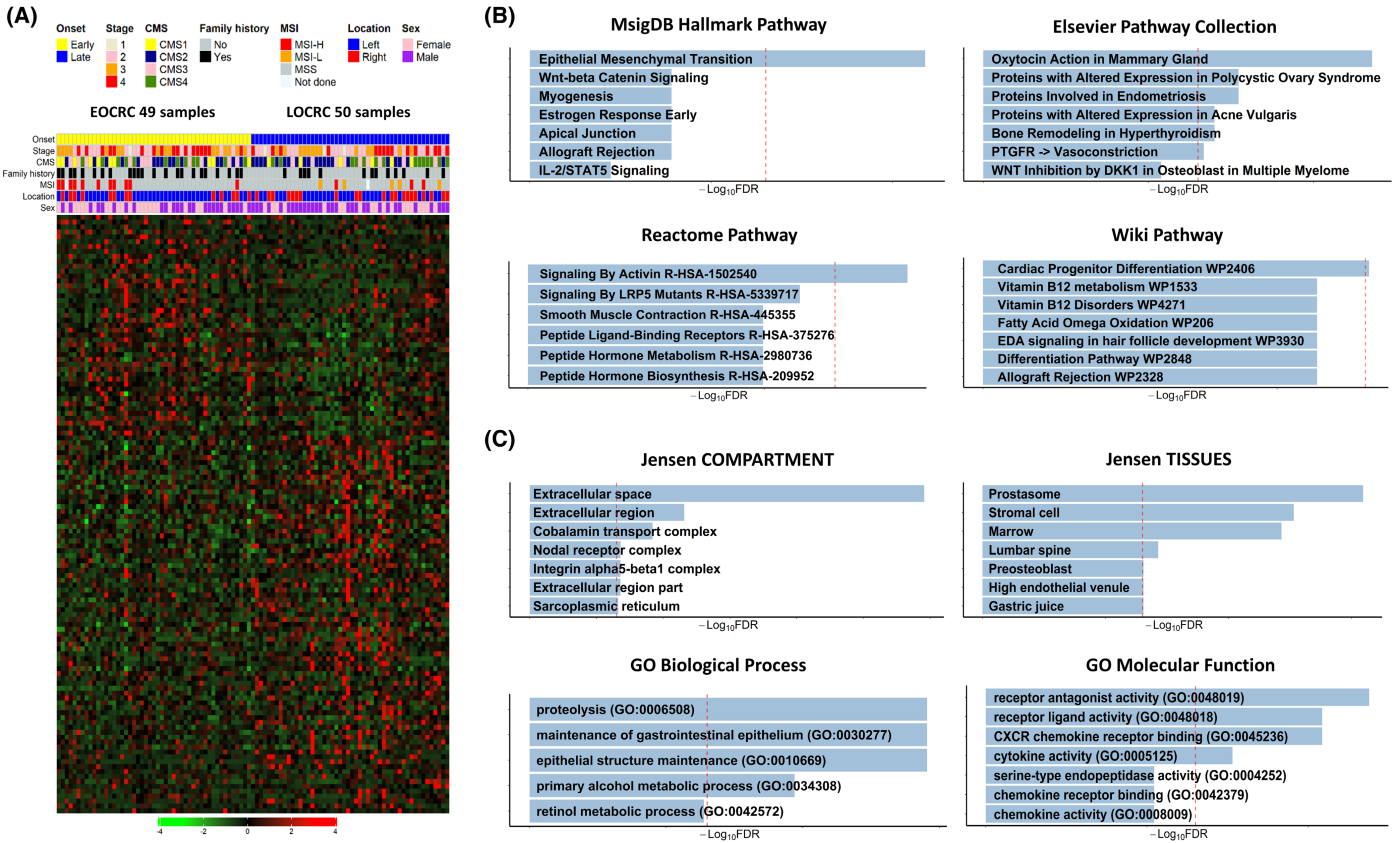
Visualization of differentially expressed genes (DEGs) identified between early‐onset colorectal cancer (EOCRC) and late‐onset colorectal cancer (LOCRC). (A) DEG heatmap. Genes were sorted in accordance with their fold‐change values. The top and bottom rows represent upregulated and downregulated genes in the EOCRC patients, respectively. (B) Pathway terms associated with the DEGs. (C) Gene ontology terms associated with the DEGs.

Our RNA sequencing data of our discovery cohort identified DEGs between the 49 tumor tissues derived from EOCRC patients and 50 from LOCRC patients (Figure 1A). We performed pathway and gene ontology analyses using these selected genes (45 upregulated and 77 downregulated genes) (Appendix S1). The epithelial‐mesenchymal transition (EMT) pathway was found to be significantly enriched (Figure 1B), and the identified gene ontologies were ECM‐related terms, epithelial structure maintenance, and immune‐related terms (Figure 1C).

### 3.3 Validation of the DEGs in the TCGA dataset and the validation cohort using real‐time RT‐PCR

**FIGURE 2:**
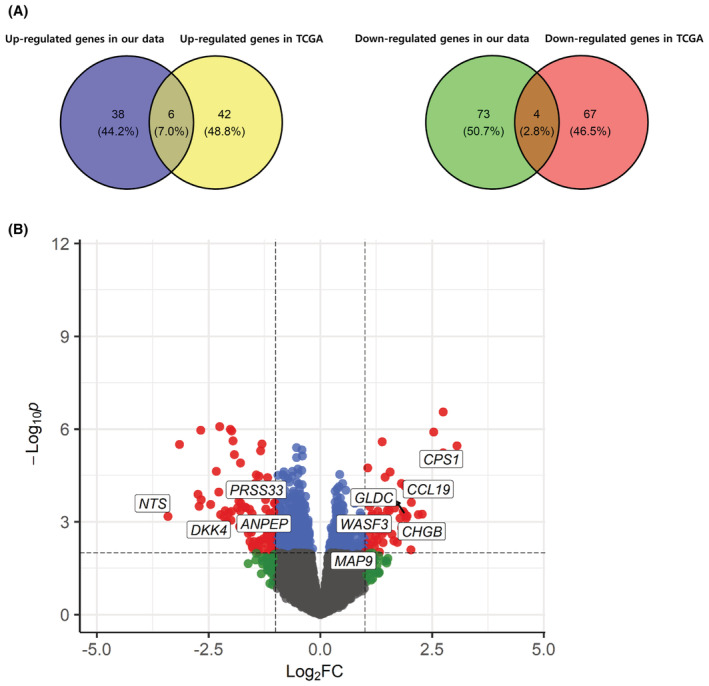
Common differentially expressed genes (DEGs) between early‐onset colorectal cancer and late‐onset colorectal cancers in the TCGA dataset. (A) Venn diagram of shared genes between the public TCGA dataset and present study cohort. (B) Volcano plot of the 10 common DEGs.

**FIGURE 3:**
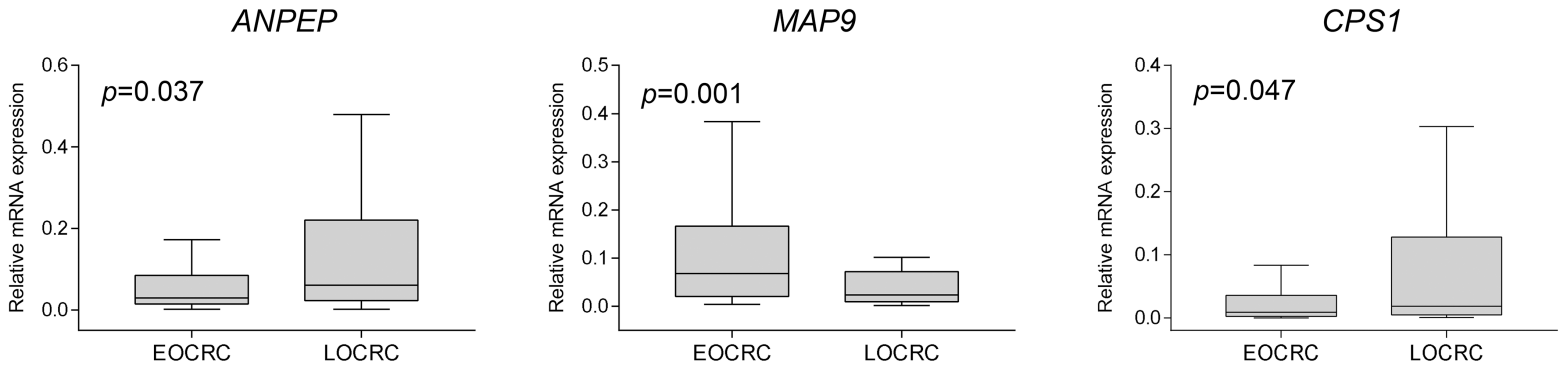
The mRNA expression of three significant DEGs in the validation cohort by real‐time RT‐PCR. Data were obtained from at least three independent experiments and values are median ± SD.

To identify EOCRC‐specific gene expression, we first selected DEGs between the EOCRCs and LOCRCs in both our discovery cohort and the TCGA cohort. To validate these DEGs, we selected common DEGs between our dataset and the TCGA datasets (Figure 2A). Ten genes (ANPEP, CCL19, CHGB, CPS1, DKK4, GLDC, MAP9, NTS, PRSS33, WASF3) showed common expression patterns in both cohorts (Figure 2B, Tables 4 and 5). We further validated these 10 genes by real‐time RT‐PCR analysis of an validation cohort (72 EOCRC and 43 LOCRC cases) to identify potential candidate biomarkers (Table 6). The ANPEP (*p* = 0.037), MAP9 (*p =* 0.0012) and CPS1 (*p* = 0.047) genes were significantly differential mRNA expression in this validation cohort (Figure 3). CPS1 showed significant down‐regulation by real‐time RT‐PCR but was found to be up‐regulated in the RNA‐seq data. The other seven genes showed no significant differences of mRNA expression in the validation cohort (Figure S1).

**TABLE 4:** Commonly upregulated genes in patients with early‐onset colorectal cancer.

Gene	Fold change (log2)	*p*‐Value	FDR	Full name
CPS1 (in TCGA)	3.05	<0.0001	0.005	Carbamoyl‐phosphate synthase 1
	1.75	<0.0001	0.003	
CCL19 (in TCGA)	2.03	0.0002	0.038	C‐C Motif chemokine ligand 19
	1.07	0.0003	0.017	
CHGB (in TCGA)	1.92	0.0007	0.056	Chromogranin B
	3.10	<0.0001	<0.0001	
GLDC (in TCGA)	1.88	0.0005	0.050	Glycine decarboxylase
	1.17	0.0002	0.013	
WASF3 (in TCGA)	1.54	0.0004	0.047	WASP family member 3
	1.15	<0.0001	0.0007	
MAP9 (in TCGA)	1.08	0.0069	0.128	Microtubule associated protein 9
	1.10	<0.0001	0.0001	

**TABLE 5:** Commonly downregulated genes in patients with early‐onset colorectal cancer.

Gene	Fold change (log2)	*p*‐Value	FDR	Full name
NTS (in TCGA)	−3.40	0.0006	0.053	Neurotensin
	−1.97	0.0016	0.055	
DKK4 (in TCGA)	−2.15	0.0002	0.053	Dickkopf WNT signaling pathway inhibitor 4
	−1.47	0.0033	0.087	
ANPEP (in TCGA)	−1.78	0.0007	0.047	Alanyl aminopeptidase, membrane
	−1.08	0.0021	0.067	
PRSS33 (in TCGA)	−1.77	0.0005	0.039	Serine protease 33
	−1.96	<0.0001	0.0006	

**TABLE 6:** Validated genes by real time RT‐PCR analysis of an validation cohort.

Gene	Fold change (log2)	*p*‐Value	FDR	Full name
ANPEP (in TCGA)	−1.78	0.0007	0.047	Alanyl aminopeptidase, membrane
	−1.08	0.0021	0.067	
MAP9 (in TCGA)	1.08	0.0069	0.128	Microtubule associated protein 9
	1.10	<0.0001	0.0001	

### 3.4 Validation of ANPEP expression at the protein level

Among two significant genes, reduced ANPEP expression was selected to be validated at protein levels by Western blotting. ANPEP protein expression was assessed in randomly selected tumor tissues from 12 CRC patients (six patients each EOCRC and LOCRC) in the validation cohort. The level of ANPEP showed significantly lower expression in tissues of EOCRC than in LOCRC (*p* = 0.027) (Figure S2).

### 3.5 Comparing ANPEP expression in CRC tumors and in normal tissue

**FIGURE 4:**
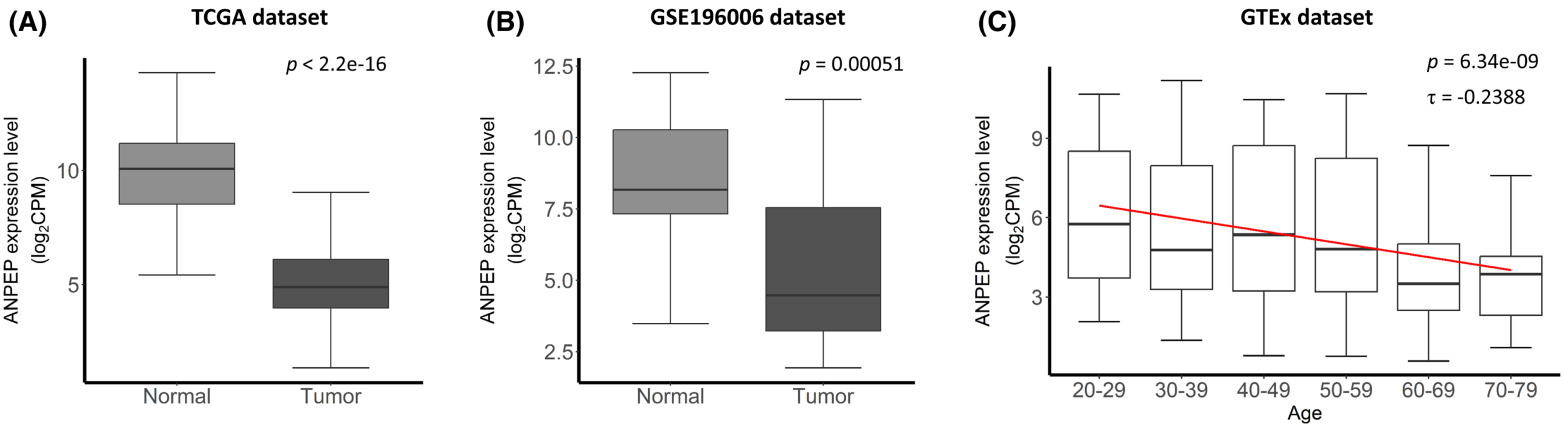
Alanyl aminopeptidase (ANPEP) expression in normal tissues in the TCGA, GSE132024, and GTEx datasets. (A) ANPEP expression in normal and tumor tissues in the TCGA dataset. ANPEP was downregulated in the tumor tissues. (B) ANPEP expression in tumor tissues of early‐onset colorectal cancer (EOCRC) and its matched pair normal tissues in the GSE132024. ANPEP has lower expression in the tumor tissues of EOCRC than its matched pair normal tissues. (C) Boxplot of ANPEP expression and patient age in normal colon tissue from the GTEx dataset. ANPEP expression decreased with age in the normal colon tissue.

ANPEP mRNA expression was constantly significant in our comparisons of the EOCRC and LOCRC samples including protein levels. Moreover, the down‐regulation of ANPEP was not only significant in the discovery cohort, TCGA cohort, and validation cohort but also in comparisons between the normal and tumor tissues (Figure 4). We first verified that ANPEP expression was lower in tumor tissues than in normal tissues in the TCGA dataset (Wilcoxon test, *p* < 2.2 × 10^−16^) (Figure 4A). A decreased ANPEP expression level was thus found to be characteristic of cancerous tissues. We then validated the ANPEP expression in the GSE196006 dataset comprised of 21 EOCRC samples and its matched normal tissues. When comparing EOCRCs with its adjacent normal tissues, ANPEP expression showed the significantly lower levels in EOCRCs than its adjacent normal tissues (Wilcoxon signed‐rank test, *p* = 0.00051) (Figure 4B). To ensure whether the reduced ANPEP is a signature of EOCRC or early age colon tissue, we verified that ANPEP expression was not decreased at a younger age when examining normal colon tissues from the GTEx dataset. In this analysis, ANPEP expression decreased gradually in normal tissues by age (Kendall's rank correlation, *p* = 6.34 × 10^−9^, coefficient τ = −0.2388) (Figure 4C). Hence, the lower level of ANPEP expression at a young age was detected only in the tissues of EOCRC, not in the tissues of normal colon.

### 3.6 Survival curves

The reduced level of ANPEP was thus found to be a significant expression pattern in EOCRC. To evaluate the possible prognostic value of this, we performed survival analysis using the human protein atlas, which contains ANPEP expression data and clinical information in the TCGA‐COAD and TCGA‐READ datasets. Both the TCGA‐COAD and TCGA‐READ exhibited that the group having a lower expression of ANPEP showed the tendency of a reduced survival rate in CRC patients (Figure S3A,B). The *p*‐value determined by log‐rank test in the survival analysis was 0.056 in TCGA‐COAD (Figure S3A) and 0.05 in TCGA‐READ (Figure S3B).

### 3.7 IPA network analysis

**FIGURE 5:**
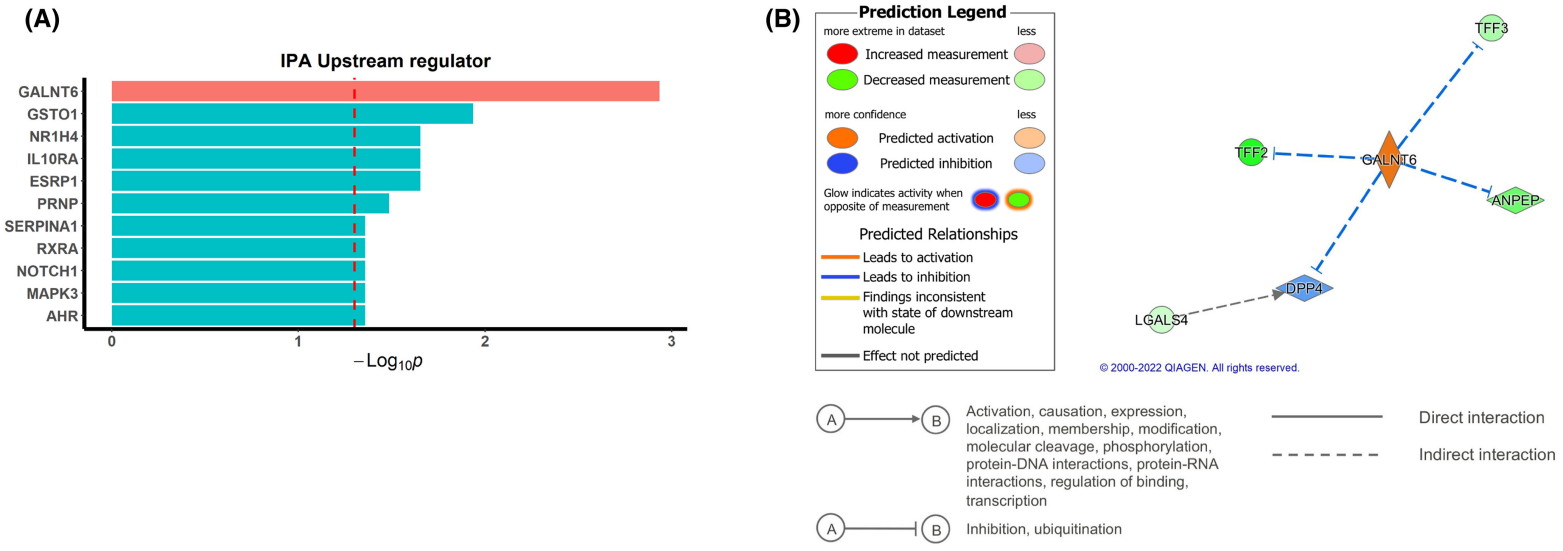
Ingenuity pathway analysis (IPA) to identify upstream regulators of alanyl aminopeptidase (ANPEP). (A) List of identified regulators, including GALNT6. (B) IPA analysis prediction that the activation of GALNT6 downregulates ANPEP.

IPA interaction network analysis was used to predict upstream regulators of ANPEP from our RNA sequencing data of our discovery cohort. Among these identified factors, GALNT6 showed an interaction with ANPEP (*p* = 0.001), regulating the inhibition of ANPEP in EOCRC patients of our discovery cohort (Figure 5A). GALNT6 also inhibits the trefoil factor family peptides, TFF2 and TFF3, which were downregulated in our EOCRC patients (Figure 5B).

### 3.8 Methylation status of the ANPEP gene

**FIGURE 6:**
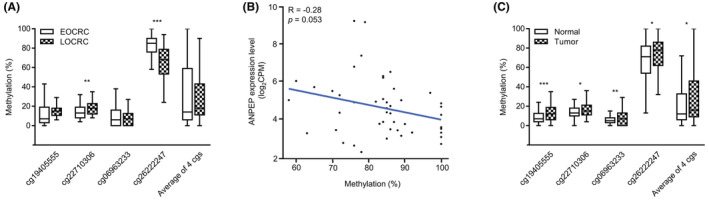
Methylation status of four probes in the promoter region of the alanyl aminopeptidase (ANPEP) gene in our discovery cohort (*p < 0.05; **p < 0.01; ***p < 0.001). (A) Methylation status of the probes between the early‐onset colorectal cancer (EOCRC) (*n* = 48) and late‐onset colorectal cancer (LOCRC) (*n* = 48) samples. The cg26222247 site was significantly hypermethylated in EOCRC compared to LOCRC. (B) Correlation between the methylation of cg26222247 and ANPEP expression in EOCRC. (C) Methylation status of the probes between normal (*n* = 96) and tumor (*n* = 96) tissues. All four probes were hypermethylated in tumor tissues compared to normal tissues.

After we confirmed that reduced ANPEP expression was a robust biomarker in EOCRC, we evaluated whether its expression level was associated with DNA methylation. We first analyzed the Illumina 450 K methylation array of the TCGA datasets. We confirmed that the ANPEP gene has 11 sites of methylation probe, except for SNP sites. Among these 11 sites, four were in CpG island and located up to 1500 bp upstream of ANPEP's transcript start site (TSS) regarded as a promoter region of ANPEP. The cg26222247 probe, one of these four sites, was found to be hypermethylated in tumor tissue compared with normal tissue, and its methylation level correlated negatively with the ANPEP expression level (*R* = −0.21, *p* = 0.00011; Figure S4). Hence, we conducted a validation of the CpG sites within the promoter region of ANPEP, including the cg26222247 site in our discovery cohort via pyrosequencing. The validation showed that the cg26222247 in tumor tissues has significantly hypermethylated in EOCRC than LOCRC (Figure 6A). In the correlation analysis between the hypermethylation of cg26222247 and ANPEP expression, our present results showed a tendency toward a negative correlation in the EOCRC cases of our discovery cohort (*R* = −0.28, *p* = 0.053; Figure 6B). The comparisons of the methylation status between the normal and tumor tissues in our current series showed that it was significantly higher for all four probes in the tumor tissues (Figure 6C).

## 4 DISCUSSION

Our current analyses suggest that reduced ANPEP expression is a robust biomarker of EOCRC as it showed constantly lower expression in our discovery cohort, a TCGA cohort, and an additional validation cohort. We first identified three genes CPS1, MAP9, and ANPEP that were found to be commonly expressed in our discovery cohort and the TCGA cohort. To assess these genes as possible biomarkers, we validated their expression in our validation cohort using real‐time RT‐PCR. Among these three genes, CPS1 expression showed opposite patterns between the discovery and validation cohorts, suggesting possible contamination of the tissues due to a lack of microdissection, an unstable expression, or differences in the exact copy number. The MAP9 gene showed a significantly consistent expression pattern in the cohorts, but its high expression could not be determined to contribute to tumorigenesis as the tumor tissues in the TCGA cohort had a lower expression than the normal tissues. By contrast, ANPEP expression was not just consistently and significantly lower in EOCRC samples compared with LOCRC samples, including Western blot analysis, but also in comparisons between the tumor and normal tissues. The ANPEP level was found to be significantly lower in EOCRCs than its paired normal tissues, and this also showed a tendency toward poor survival. We thus speculated that ANPEP may play a tumor‐suppressive role in EOCRC. Previous studies have also indicated that ANPEP expression is significantly lower in CRCs than in normal tissues and that CRC patients with lower ANPEP activity in their tumors had poorer overall survival.^32^, ^33^ Moreover, a low expression of ANPEP has been reported as a target associated with malignant transformation and tumor cell invasion in CRC.^34^ In addition, since EOCRC cases show more frequent lymphovascular and venous invasion, younger CRC patients tend to have a higher rate of metastasis.^35^, ^36^ These aggressive phenotypes, therefore, need to be further verified and it will be important to ascertain whether they have an association with the large extracellular carboxyterminal domain of ANPEP containing a pentapeptide consensus sequence of the zinc‐binding metalloproteinase superfamily. Our present findings and previously reported data thus indicate that ANPEP expression may affect the malignant and aggressive potential of EOCRC.

ANPEP is an enzyme that functions in glutathione (GSH) metabolism. Although this metabolism is well known for its antioxidant impacts, it has been further associated with detoxification and inflammation in the colon. Some studies have reported an association between CRC and inflammatory bowel disease with an imbalance or low function of GSH metabolism.^37^, ^38^, ^39^ Further, previous research has also demonstrated that GSH metabolism is a significant pathway in early‐onset, sporadic CRC.^40^ However, it is unknown how the GSH level affects EOCRC regarding the enzymatic function of ANPEP. Another previous research has reported that GSH metabolism and low expression of ANPEP were associated with the induction of EMT in non‐small cell lung cancer (NSCLC).^41^ Interestingly, our result showed that the EMT pathway was a significantly different pathway between EOCRCs and LOCRCs (Figure 1B). To clarify this result, further research should be conducted on whether GSH metabolism and low expression of ANPEP are associated with the induction of EMT in EOCRC.

Recently, a previous EOCRC study based on gene expression profiling reported that the biomarker, PDGFRA, was significantly correlated with the EMT marker genes.^9^ Another recent study has reported that the biomarker of EOCRC, PEG10, that they identified played a role in tumor cell invasion.^10^ In terms of genomic variant, the deletion of the NOMO1 gene has been reported as a clinical marker in EOCRC, and the regulation of cell migration has been suggested for its role in tumorigenesis.^42^ In summary, our study showed concordance with those EOCRC studies that have reported EMT‐related features of the biomarker in EOCRC, regarding that the EMT pathway was significantly distinct between EOCRC and LOCRC in our research. In another respect, some studies have identified immune‐related gene expressions as a biomarker of EOCRC, suggesting that EOCRC was distinct in immunity compared to LOCRC.^43^, ^44^ Despite the research on EOCRC, it remains challenging to categorize EOCRC as a distinct subtype of CRC based on genomic signatures since the heterogeneity of CRC and the lack of EOCRC samples make it difficult to identify specific biomarkers and genomic pathogenesis. Although the difficulty of EOCRC research is high, these findings illuminate the malignant signature of EOCRC and provide a clue of the genomic pathogenesis of EOCRC. Future research on EOCRC may illustrate the association in both respects and develop the model for categorizing EOCRC as a subtype of CRC.

We additionally found upstream regulators of ANPEP in our IPA network analysis. This analysis predicted that GALNT6 (polypeptide N‐acetylgalactosaminyltransferase 6; GalNAc‐T6) was activated in EOCRC where it inhibited ANPEP expression. Recent studies have reported that GALNT6 is highly upregulated in CRC tissues.^45^, ^46^ In addition, GalNAc‐Ts have been reported to influence several tumorigenic processes, including immune evasion, invasion, EMT induction, and metastasis.^46^, ^47^, ^48^ Moreover, a low expression of TFF2 and TFF3, which were predicted to be inhibited by GALNT6, have been reported to associate with gastric cancer.^49^, ^50^ Of particular note, a low expression of TFF3 has been proposed to be associated with colon cancer.^51^, ^52^ Since TFF3 has a major role in protecting the intestinal barrier, colitis has been found to develop in TFF3 knock‐out mice.^53^, ^54^ Although GALNT6 was predicted to be an upstream regulator of ANPEP and TFF3, its mechanistic function in EOCRC still needs to be confirmed.

We found in our present study that ANPEP is associated with EOCRC in terms of its gene expression in tumor tissue. In another respect, EOCRC cases have a higher prevalence of hereditary cancer syndromes than LOCRC cases. The underlying genetic mechanisms, including genetic alterations, have been identified previously through the study of hereditary CRCs (HCRC) such as FAP and HNPCC. Genetic alterations in germline susceptibility genes involved in HCRC can accelerate tumorigenesis and cause EOCRC. However, since we had excluded tumor tissues of FAP and HNPCC patients meeting the Amsterdam‐I criteria, or who underwent preoperative chemo/radiotherapy, the effects of germline susceptibility genes were minimized in our present study and the expressions of those genes were not significantly different between EOCRC and LOCRC (Table 7).

**TABLE 7:** Germline susceptibility genes associated with hereditary cancer syndrome.

Gene	Fold change (log2)	*p*‐Value	FDR	Full name
MLH1 (in TCGA)	0.29	0.0074	0.1315	Mutl homolog 1
	0.21	0.0848	0.0848	
MSH2 (in TCGA)	0.04	0.7518	0.7518	Muts homolog 2
	0.12	0.1367	0.1367	
MUTYH (in TCGA)	−0.13	0.3925	0.3925	mutY DNA glycosylase
	0.06	0.5108	0.5108	
MSH6 (in TCGA)	0.10	0.3897	0.3897	mutS homolog 6
	0.08	0.2704	0.2704	
PMS2 (in TCGA)	0.04	0.7242	0.7247	PMS1 homolog 2
	0.07	0.2594	0.2594	
APC (in TCGA)	0.26	0.1151	0.4037	Adenomatous polyposis coli
	0.15	0.1737	0.6041	
SMAD4 (in TCGA)	0.43	0.0045	0.1096	Mothers against decapentaplegic homolog 4
	−0.13	0.1424	0.5600	
BRCA1 (in TCGA)	0.30	0.0369	0.2483	Breast cancer gene 1
	0.22	0.0143	0.2020	
BRCA2 (in TCGA)	0.29	0.1761	0.4887	Breast cancer gene 2
	−0.05	0.6474	0.9063	
ATM (in TCGA)	0.14	0.3108	0.6251	Ataxia telangiectasia mutated
	0.12	0.2562	0.6907	
CHEK2 (in TCGA)	0.01	0.9210	0.9721	Checkpoint kinase 2
	0.05	0.4747	0.8289	
PALB2 (in TCGA)	0.11	0.1752	0.4876	Partner and localizer of BRCA2
	0.11	0.0826	0.4519	
CDKN2A (in TCGA)	−0.64	0.0791	0.3416	Cyclin‐dependent kinase inhibitor 2A
	−0.59	0.0289	0.281	

After we identified ANPEP as a robust biomarker of EOCRC, we sought to identify the mechanistic process of reduced ANPEP expression. We first examined whether gene methylation and copy number alterations were responsible for this lower expression, using the TCGA datasets (Figure S2). We found from these analyses that the methylation of cg26222247, a site in the promoter region of ANPEP, was significantly negatively correlated with ANPEP expression. Moreover, previous finding regarding silenced ANPEP in prostate cancer have revealed that hypermethylation in the promoter region of ANPEP correlated inversely with its expression.^55^ To validate the result in our present experiments, we analyzed the methylation status of the ANPEP gene promoter region in our discovery cohort. The results showed the same tendencies in terms of the methylation patterns in the EOCRC samples, but this was not statistically significant possibly due to the sample size or other unknown factors that regulate ANPEP expression in EOCRC. With regard to our finding that ANPEP expression was reduced in EOCRC, we found that the hypermethylation of cg26222247 was also significantly higher in the EOCRC than in the LOCRC samples for both the normal and tumor tissues in our discovery cohort. However, it remains unclear whether the methylation of cg26222247 in normal tissues contributes to tumorigenesis in EOCRC, except for HCRC.

## 5 CONCLUSION

In conclusion, we have recently identified the down‐regulated expression of ANPEP as a significant marker of EOCRC and its hypermethylated site. Additionally, the EMT pathway was identified as a significant pathway of EOCRC in this study. This finding contributes to understanding EOCRC and characterizing EOCRC as a different type of CRC, distinct from LOCRC in the future.

## AUTHOR CONTRIBUTIONS

**Ye Jin Ha:** Data curation (equal); formal analysis (equal); investigation (equal); methodology (equal); project administration (lead); validation (lead); visualization (equal); writing – original draft (lead); writing – review and editing (lead). **Yun Jae Shin:** Data curation (lead); formal analysis (lead); investigation (lead); methodology (lead); validation (lead); visualization (lead); writing – original draft (lead). **Ka Hee Tak:** Data curation (equal); formal analysis (equal); methodology (equal); validation (equal); writing – original draft (equal). **Jong Lyul Park:** Data curation (supporting); investigation (supporting); methodology (supporting); project administration (supporting); supervision (supporting); writing – review and editing (supporting). **Jeong Hwan Kim:** Data curation (supporting); formal analysis (supporting); methodology (supporting); validation (supporting); writing – original draft (supporting). **Jong Lyul Lee:** Data curation (supporting); formal analysis (supporting); investigation (supporting); methodology (supporting); resources (supporting); validation (supporting). **Yong Sik Yoon:** Data curation (supporting); formal analysis (supporting); methodology (supporting); resources (supporting). **Chan Wook Kim:** Conceptualization (lead); data curation (equal); formal analysis (equal); funding acquisition (lead); methodology (equal); resources (equal); supervision (equal); writing – review and editing (equal). **Seon Young Kim:** Conceptualization (supporting); funding acquisition (lead); methodology (supporting); project administration (supporting); supervision (supporting); writing – review and editing (lead). **Jin Cheon Kim:** Data curation (equal); investigation (equal); methodology (equal); resources (equal); writing – review and editing (equal).

## CONFLICT OF INTEREST STATEMENT

The authors declare no competing financial or other interests in relation to this article.

## ETHICAL APPROVAL STATEMENT

This study was approved by the Asan Medical Center Institutional Review Board (IRB no. 2019–1367). Requirement for informed consent was waived.

## CONSENT FOR PUBLICATION

All authors have read and approved the manuscript.

## Supporting information


Appendix S1.

Figure S1.

Figure S2.

Figure S3.

Figure S4.

## Data Availability

The data sets used in this study are available from NCBI's Gene Expression Omnibus (GEO) (Accession ID: GSE213092) and Korean Nucleotide Archive (KoNA) (Accession ID: PRJKA220461) (https://www.kobic.re.kr/kona/).
