# Supplementary figures and images for: Reduced expression of alanyl aminopeptidase is a robust biomarker of non‐familial adenomatous polyposis and non‐hereditary nonpolyposis colorectal cancer syndrome early‐onset colorectal cancer
Source: Cancer Med. 2023 Feb 7;12(8):10091–104. doi: 10.1002/cam4.5675 (PMC10166950; doi:10.1002/cam4.5675)

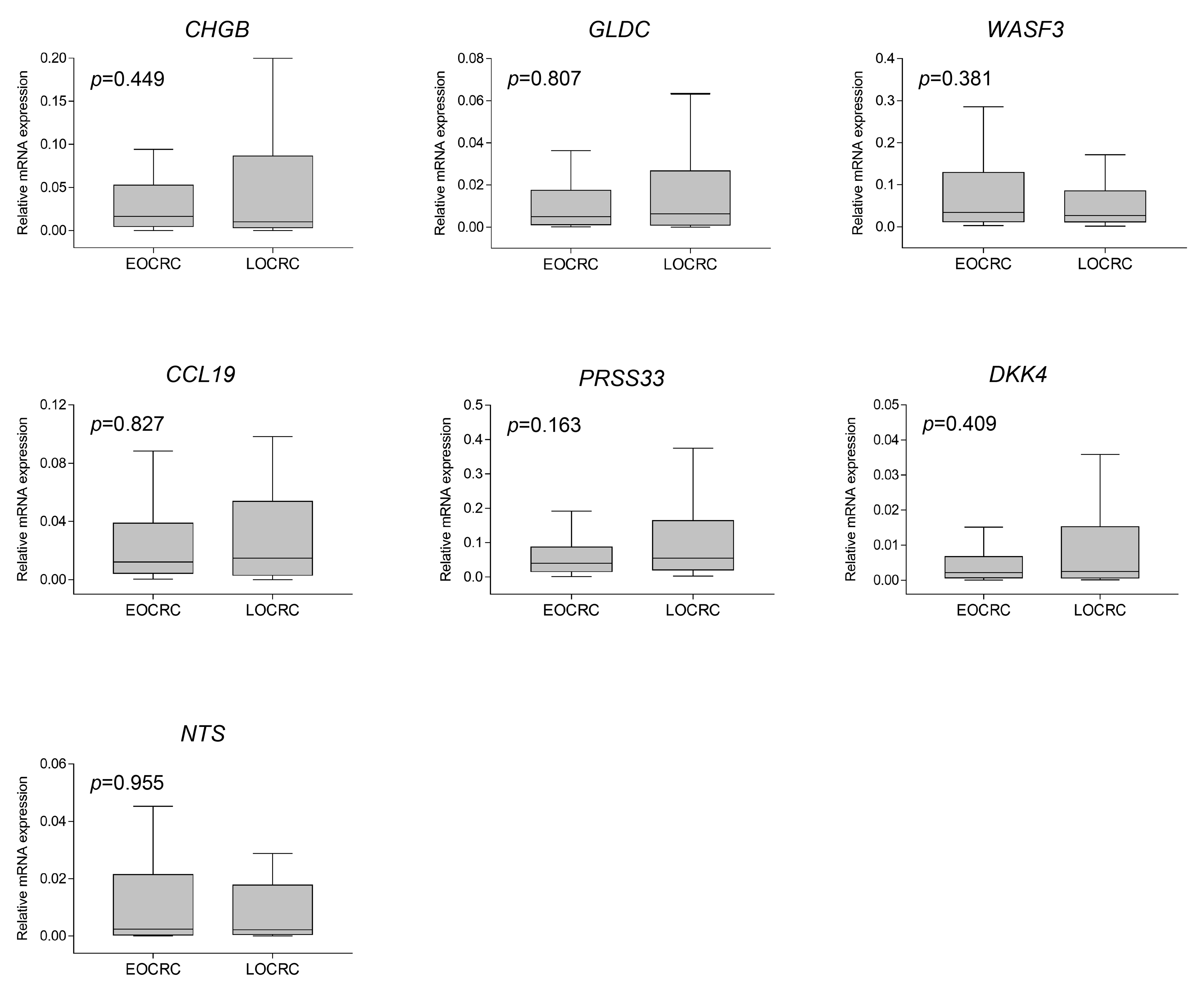

Supplement: Supplementary file 2 — Figure S1. [file CAM4-12-10091-s002.tif]

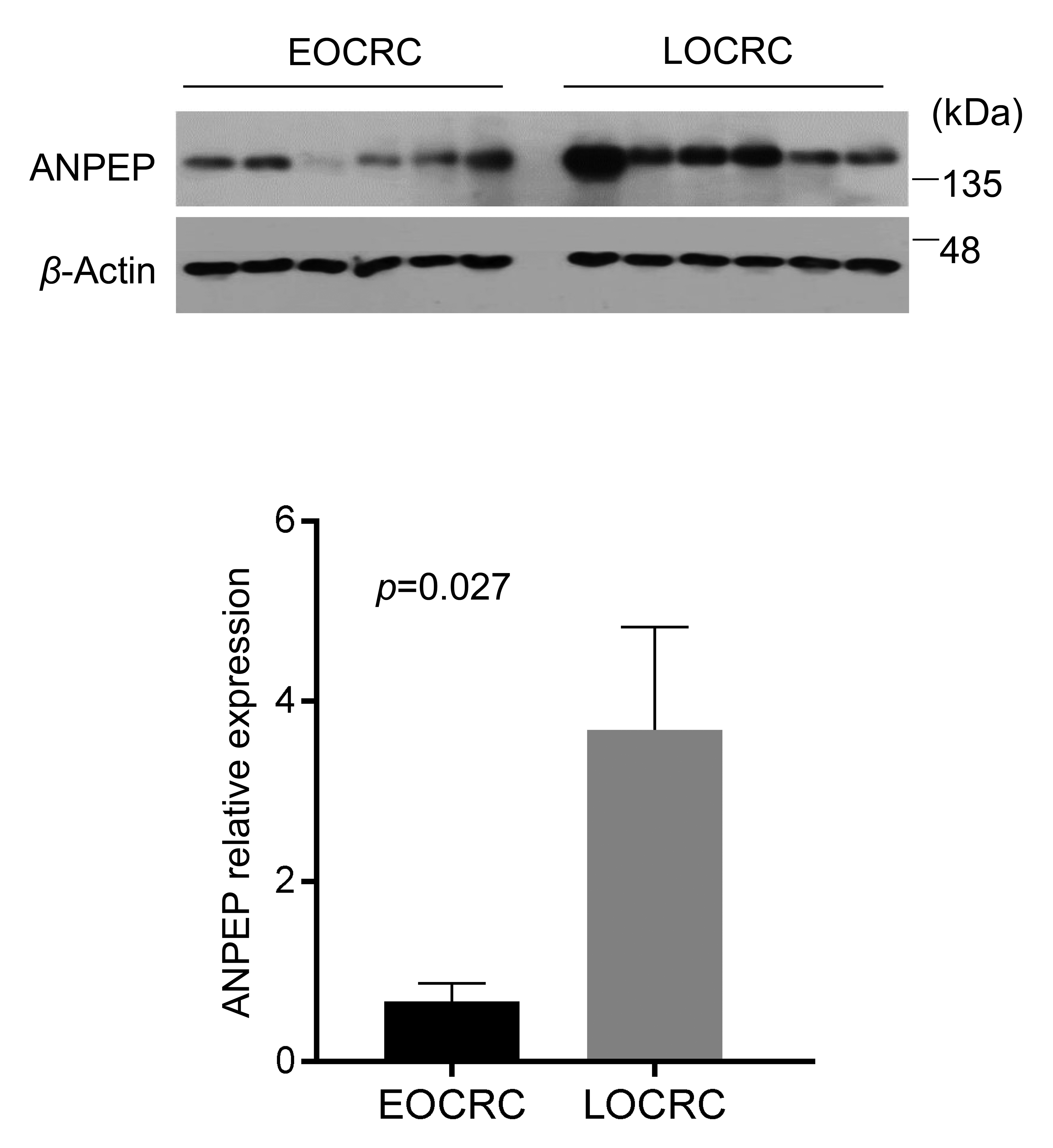

Supplement: Supplementary file 3 — Figure S2. [file CAM4-12-10091-s005.tif]

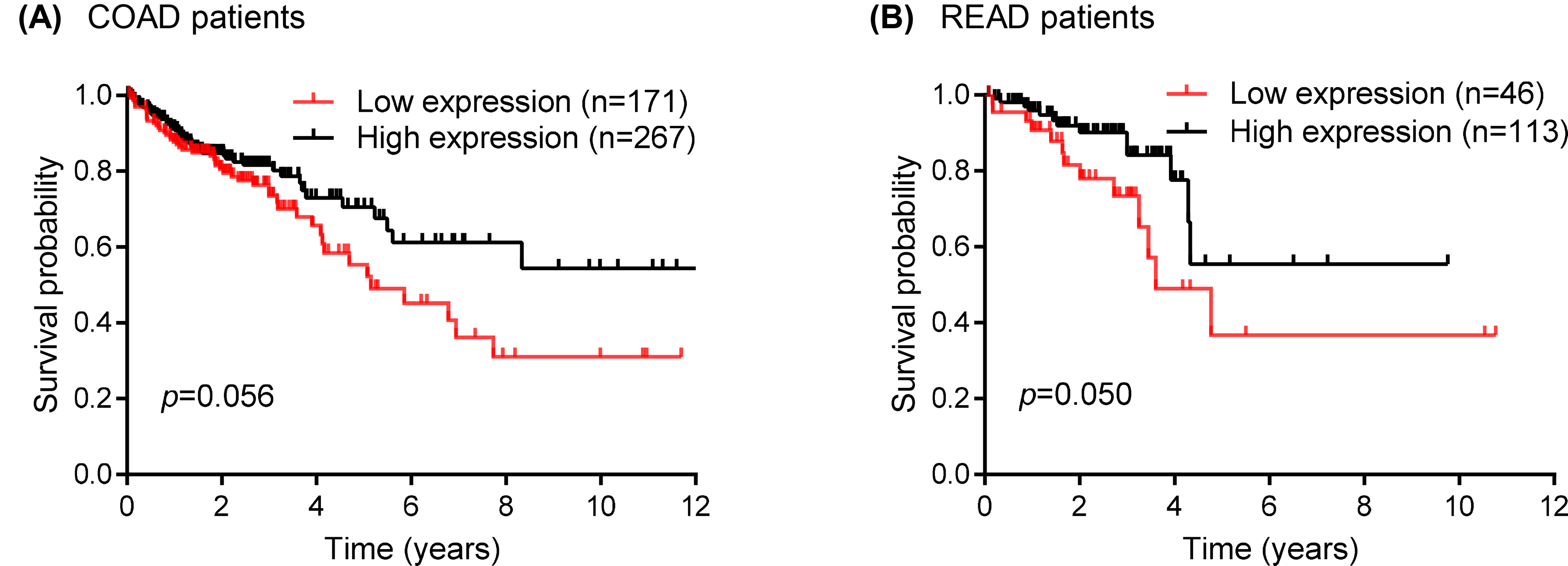

Supplement: Supplementary file 4 — Figure S3. [file CAM4-12-10091-s003.tif]

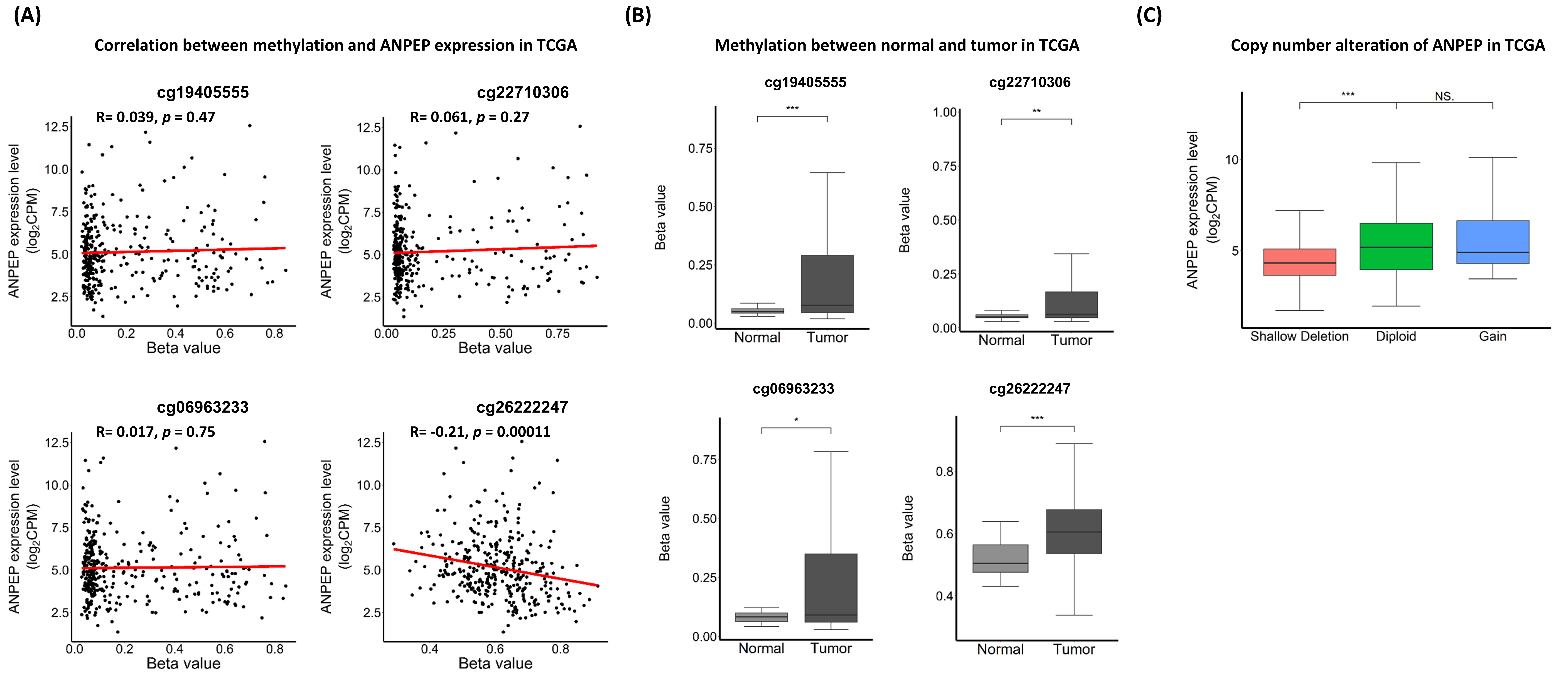

Supplement: Supplementary file 5 — Figure S4. [file CAM4-12-10091-s004.tif]
